# The iso-response method: measuring neuronal stimulus integration with closed-loop experiments

**DOI:** 10.3389/fncir.2012.00104

**Published:** 2012-12-19

**Authors:** Tim Gollisch, Andreas V. M. Herz

**Affiliations:** ^1^Department of Ophthalmology and Bernstein Center for Computational Neuroscience Göttingen, University Medical Center GöttingenGöttingen, Germany; ^2^Department Biology II and Bernstein Center for Computational Neuroscience Munich, Ludwig-Maximilians-Universität MünchenMunich, Germany

**Keywords:** neural computation, sensory systems, stimulus integration, closed-loop experiment, isoresponse, neuron models

## Abstract

Throughout the nervous system, neurons integrate high-dimensional input streams and transform them into an output of their own. This integration of incoming signals involves filtering processes and complex non-linear operations. The shapes of these filters and non-linearities determine the computational features of single neurons and their functional roles within larger networks. A detailed characterization of signal integration is thus a central ingredient to understanding information processing in neural circuits. Conventional methods for measuring single-neuron response properties, such as reverse correlation, however, are often limited by the implicit assumption that stimulus integration occurs in a linear fashion. Here, we review a conceptual and experimental alternative that is based on exploring the space of those sensory stimuli that result in the *same* neural output. As demonstrated by recent results in the auditory and visual system, such *iso-response stimuli* can be used to identify the non-linearities relevant for stimulus integration, disentangle consecutive neural processing steps, and determine their characteristics with unprecedented precision. Automated closed-loop experiments are crucial for this advance, allowing rapid search strategies for identifying iso-response stimuli during experiments. Prime targets for the method are feed-forward neural signaling chains in sensory systems, but the method has also been successfully applied to feedback systems. Depending on the specific question, “iso-response” may refer to a predefined firing rate, single-spike probability, first-spike latency, or other output measures. Examples from different studies show that substantial progress in understanding neural dynamics and coding can be achieved once rapid online data analysis and stimulus generation, adaptive sampling, and computational modeling are tightly integrated into experiments.

## Introduction

Mapping high-dimensional input streams into low-dimensional output spike trains is a core operation of almost every neuron in the brain. No auditory neuron is sensitive to only one frequency of a time-varying sound signal, no visual neuron responds to only one wavelength in a light stimulus. Both types of neurons rather integrate inputs over a range of frequencies. Similarly, strong dimensional reduction also occurs when retinal ganglion cells integrate signals over space via tens to hundreds of bipolar cells with smaller receptive fields, when pyramidal cells combine input from 10,000 other cortical neurons, or when cerebellar Purkinje cells are innervated by 200,000 parallel fibers to cause well-orchestrated movement patterns. In all these cases, huge amounts of information are lost—and need to be lost, or rather discarded, so that those particular stimulus combinations can be distilled that are indeed important for behavior.

Extracting the specific rule of how a given neuron combines its inputs is a prerequisite for understanding its computational function. Consider, for example, the responses of auditory neurons to a sound pressure wave s(t) with several frequency components, s(t) = Σ_i_ s_i_ cos(2πν_i_t). A neuron whose firing rate *r* is some function *f* of the summed amplitudes, *r* = *f* (Σ_i_s_i_), encodes the maximal sound amplitude whereas another neuron whose activity depends on the summed squares of these components, *r* = *g* (Σ_i_s^2^_i_), encodes sound energy. In both cases, it is a particular scalar quantity, Σ_i_s_i_ or Σ_i_s^2^_i_, respectively, that matters for the neuron's firing rate, whereas the detailed composition of the vector (s_1_, s_2_, s_3_,…) is irrelevant. Similarly, the shapes of the output non-linearities *f* and *g* are of no importance for the fact that the two neurons encode sound amplitude and energy, respectively, as long as the cells' firing thresholds, saturation levels, and input sensitivities are such that behaviorally important signal ranges can be encoded. Moreover, this simple example demonstrates that measuring a cell's input-output relation by changing the total input strength—as often done in electrophysiological experiments—will provide information about the output non-linearity, but will typically *not* reveal which computation is represented by the cell's activity.

This observation calls for alternative methods to investigate the principles and mechanisms of stimulus integration and to reveal the potential non-linearities involved in this process. Here, we review recent advances to this end, based on closed-loop measurements of iso-response stimuli. Iso-response stimuli are defined as those combinations of the individual stimulus components that yield the same predefined neuronal response. To efficiently search for sets of such stimulus combinations in neurophysiological experiments, closed-loop experiments with automated data analysis and appropriate feedback to the applied stimulation provide an essential ingredient. As discussed and exemplified below, this iso-response approach has already led to new fundamental insights into the function of neurons and neural circuits in different sensory modalities and provides a large potential for future developments and advances in a wide range of systems.

## Model framework for investigating stimulus integration

A common methodology for analyzing a neuron's stimulus-response relation is based on system identification theory and applies the framework of cascade models (see e.g., Marmarelis and Marmarelis, 1978; Korenberg and Hunter, 1986). These models aim at describing input-output systems in a phenomenological way by a sequence of mathematical primitives, such as linear filters and non-linear transformations. The most prominent member of the cascade model family is arguably the LN model (Hunter and Korenberg, 1986; Sakai, 1992; Meister and Berry, 1999; Chichilnisky, 2001; Paninski, 2003; Schwartz et al., 2006), which comprises a stage of linear filtering of the stimulus, followed by a non-linear transformation of the filter output.

The appeal of this model stems from its simple interpretation; the linear filter describes how different stimulus components are integrated and thus represents the neuron's receptive field structure, whereas the non-linearity captures the output transformation induced by spike generation. In addition, the model elements can be derived in physiological experiments with relative ease. The linear filter, for example, can readily be found through calculating the spike-triggered average (STA) in response to broad-band stimulation, such as white-noise input (de Boer and Kuyper, 1968; Bryant and Segundo, 1976; Eggermont et al., 1983; Chichilnisky, 2001; Paninski, 2003). In using a single linear filter for the stimulus integration stage, however, the LN model implicitly assumes that the entire stimulus integration occurs in a linear fashion. All non-linear effects are relegated to the output non-linearity. The LN model is thus of limited use as soon as the true processing chain contains non-linear operations before stimulus integration is complete. A prominent example are complex cells in visual cortex, whose input stage corresponds to the sum of two squared Gabor filter signals—resulting in the well-known energy model (Adelson and Bergen, 1985)—so that the cells' input-output function corresponds to an LNLN instead of an LN cascade.

A step forward is made by analyzing the spike-triggered covariance (STC) matrix (Bryant and Segundo, 1976; de Ruyter van Steveninck and Bialek, 1988; Brenner et al., 2000; Schwartz et al., 2006; Samengo and Gollisch, 2012), an extension of the STA. STC analysis allows one to extract multiple linear filters whose contributions are non-linearly combined. This works well for assessing whether a neuron can be described as a linear integrator (STC then yields just one filter) or is better described by non-linear stimulus integration (STC yields multiple filters). Furthermore, this analysis can thereby identify those stimulus components (i.e., filters) whose non-linear integration underlies a neuron's response characteristics. Yet, STC analysis by itself is typically not sufficient for quantitatively assessing the functional form of non-linear stimulus integration, in particular because several parallel filters have to be considered and non-linear effects of stimulus integration and of the output stage need to be separated. We will return to this aspect later and discuss the complementary nature of STC and iso-response analysis.

**Figure 1:**
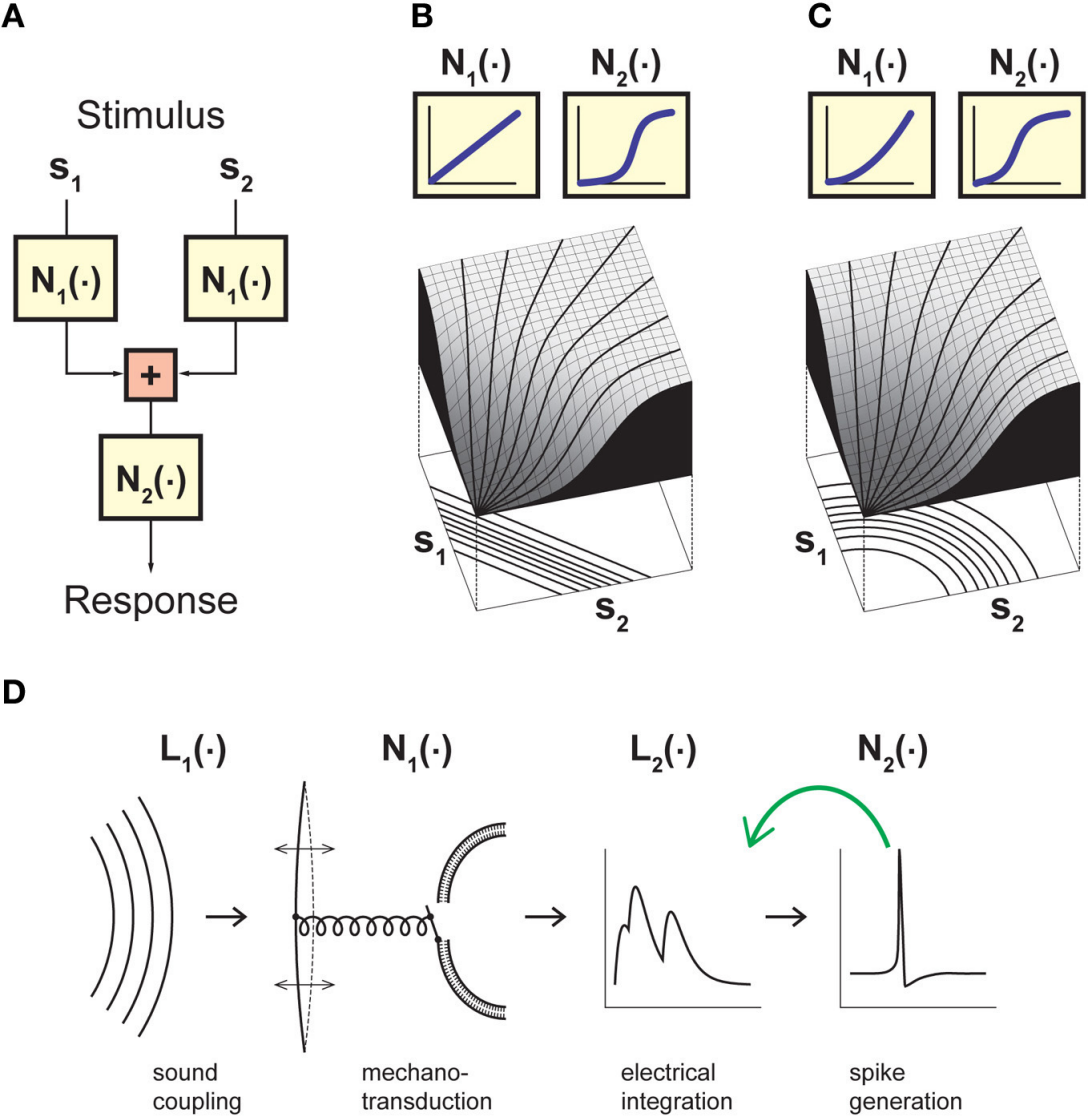
**Model framework for analyzing neuronal stimulus integration with iso-response measurements. (A)** A canonical model for separating non-linear effects before and after stimulus integration. The model considers two (or more) separate inputs s_1_…s_n_, which each undergo a non-linearity N_1_ before summation, and a final output non-linearity N_2_. Stimulus integration is governed by the non-linearity N_1_, whereas N_2_ provides a transformation of the integrated signal. Assessing the nature of stimulus integration thus amounts to measuring N_1_ independently of N_2_. **(B)** Model responses obtained with linear stimulus integration and sigmoidal output transformation. The response surface is dominated by the sigmoidal shape of the output non-linearity, but the iso-response curves shown below the surface plot are straight lines and thus reveal the linear nature of stimulus integration in this model. **(C)** Same as **(B)**, but with a quadratic non-linearity N_1_ and thus non-linear stimulus integration. While the response surface is still dominated by a sigmoidal shape, the iso-response curves are now circles and thus reveal the quadratic non-linearity relevant for stimulus integration. The non-linearities N_2_ in **(B)** and **(C)** were adjusted such that if s_1_ or s_2_ are presented alone, the input-output relations are identical in the two models. **(D)** A cascade model for sound transduction, consisting of two linear filters L_1_ and L_2_ and two non-linear transformations N_1_ and N_2_. The green arrow indicates that the cascade may also include a feedback mechanism, corresponding to adaptation induced by the neuron's spiking activity. Panels **(B)** and **(C)** adapted from Gollisch and Herz (2003) with permission, Copyright (2003), Frankfurt Institute for Advanced Studies. Panel **(D)** adapted from Gollisch and Herz (2005) according to the Creative Commons Attribution License.

Given the above considerations, let us thus consider a model that goes beyond the LN model by incorporating an explicit separation between non-linear operations before and after stimulus integration has taken place (Figure 1A). The input to this model is provided by two or more stimulus components s_1_, …, s_n_ that separately undergo some non-linear transformation N_1_(·). The linear sum of these terms then serves as input to a second non-linearity N_2_(·). This results in a sequence of non-linear, linear, and again non-linear operations and is thus correspondingly called an NLN cascade (Marmarelis and Marmarelis, 1978; Korenberg and Hunter, 1986). In what follows, the NLN cascade model serves as a canonical framework for studying stimulus integration and helps us formalize the relevant challenges and strategies. More complex cascades can be obtained by extending the linear sum to a linear filter operation or by combining more elementary building blocks. For example, auditory signal transduction has been described by an LNLN cascade (Figure 1D; Gollisch and Herz, 2005).

The important feature of the canonical model of Figure 1A is that it separates non-linear transformations occurring after stimulus integration has taken place (function N_2_) from non-linear transformations occurring just before or in the course of stimulus integration (function N_1_). Thus, it is the function N_1_ that determines the nature of stimulus integration and dictates which scalar measure is distilled out of the combination of stimulus components s_i_. N_2_, on the other hand, provides a transformation that determines how this scalar measure is represented, but does not affect what is represented in the neuron's output. Hence, the benefit of the canonical model of Figure 1A is to provide a framework for separating non-linearities that are relevant for stimulus integration from those that are irrelevant for this purpose, even if they strongly influence the neural output, for example in the form of an all-or-none spike generation threshold or pronounced response saturation.

## The Iso-response method

As seen in the discussion above, a fundamental challenge to studying neuronal information processing is that non-linearities relevant for stimulus integration need to be separated from subsequent non-linearities, in particular those at the output stage. To approach this challenge, an experimental design is needed that directly reflects these different non-linear processing stages. Crucial insight is provided by a strategy known from measuring threshold curves in neurobiology (Evans, 1975) or using equivalence criteria in psychophysics (Jameson and Hurvich, 1972): instead of estimating the full input-output relation, stimulus parameters are varied such that the neuron's response stays at a *constant level* (Gollisch et al., 2002; Gollisch and Herz, 2003).

The key idea behind this concept is that staying at a constant response level removes the effect of the output non-linearity in the canonical model of stimulus integration (Figure 1A). How different stimulus components have to be combined to reach this response level thus serves as a direct signature of the nature of stimulus integration. This is most easily seen when considering a system with two independent input channels s_1_ and s_2_. In the two-dimensional stimulus space spanned by s_1_ and s_2_, iso-response stimuli are typically located on one-dimensional curves, which we call iso-response curves. Linear integration, for example, is characterized by iso-response curves that are straight lines, even if the overall response function of the neuron is strongly non-linear because of the output non-linearity (Figure 1B). Deviations from linearity in the integration process, on the other hand, lead to differently shaped curves. As a simple example, integration in the form of a sum of squares yields circular iso-response curves, defined by the circle equation s^2^_1_ + s^2^_2_ = const (Figure 1C).

In higher dimensional stimulus spaces, the iso-response curves become iso-response manifolds. Linear integration then corresponds to an iso-response manifold whose shape is a hyperplane. The iso-response manifolds represent the invariances of a neuron's input-output relation and therefore provide an important characterization of the neuron's computational role, even when considering only low-dimensional stimulus subspaces. These still supply a signature of the neuron's invariances; for example, if a neuron has ellipsoids as iso-response manifolds in a high-dimensional stimulus space, an investigation of a two-dimensional planar subspace will display elliptic iso-response curves. High-dimensional hyperplanes, on the other hand, always yield simple straight lines in a two-dimensional projection.

The prime advantage of the method lies in the fact that the iso-response manifolds are independent of the potentially highly non-linear operation occurring at the final output stage; the iso-response approach relies solely on comparisons of stimuli for which this output stage has identical effects. This focus on a particular response range also makes the approach experimentally efficient, which is of special importance when data acquisition time is limited. Furthermore, by their very definition, iso-response stimuli are “perceived” as identical by the neuron under investigation. The shape of an iso-response manifold thus has a direct functional interpretation, whereas it is often difficult to assign a particular meaning to the specific shape of a neuron's traditional stimulus-response curve. Finally, as the full stimulus-response curve need not be determined within the iso-response paradigm, strong stimulation can be obviated so that experimental artifacts caused by activity-dependent cellular fatigue are not an issue.

Depending on the investigated neuron or on the considered stimuli, different neuronal output characteristics may be relevant for information transmission. Accordingly, the iso-response concept is not limited to “iso-firing rate” but can also be implemented as “iso-first-spike latency” (Bölinger and Gollisch, 2012), “iso-firing phase,” or other iso-response variants. In fact, *every* neural response feature that depends on input stimuli can serve as an iso-response dimension, including the value of the probability that a single spike occurred at all (Gollisch and Herz, 2005). Other useful target response measures could be the firing phase relative to some underlying large-scale rhythm or a specific temporal discharge pattern. This goes along with a freedom of choice regarding the dynamics of the chosen stimulus. Iso-response methods can be applied with extremely brief, highly non-stationary stimuli down to the sub-millisecond range (Gollisch and Herz, 2005) as well as with longer, stationary stimuli (Gollisch et al., 2002; Horwitz and Hass, 2012). The first paradigm provides a chance to disentangle rapid biophysical processes that subserve temporally precise stimulus integration, whereas the second setting allows one to focus on the system's spectral or spatial integration properties, independently of temporal dynamics. Furthermore, a given neuron may use different coding schemes for different stimulus attributes. To cover such multiplexing of information (or rule it out for the neuron under study), one can apply different iso-response measures within one experiment (Bölinger and Gollisch, 2012).

## Historical background of Iso-response measurements

The concept of measuring different stimuli that yield the same response also underlies the measurements of threshold tuning curves, which are widely used, for example, to characterize auditory neurons (Tasaki, 1954; Holton and Weiss, 1983; Harris and Dallos, 1984; Geisler et al., 1990). Here, the predefined response is typically set to be the minimal notable difference from baseline activity, and these thresholds are obtained along the axis of varying sound frequency. The measurements of threshold—as compared to measuring the response strengths for a given stimulus amplitude at different sound frequencies—has the advantage that it avoids overly strong stimulation, which would trigger non-linear suppression mechanisms, blurring the tuning characteristics (Eustaquio-Martín and Lopez-Poveda, 2011).

Other early applications of iso-response measurements have been carried out in the visual system. In the frog retina, threshold intensities of spots in the receptive field center of a recorded ganglion cell were obtained for different light intensity in the surround (Barlow, 1953). This was used to study whether signals in the center and surround of the receptive field were combined in a linear or non-linear fashion. For neurons in primary visual cortex, the combined direction and spatial frequency selectivity was characterized by measuring responses to different combinations of motion direction and spatial frequency and then extracting iso-response curves in the 2D direction–frequency space (Jones et al., 1987). The purpose of these iso-response curves was to provide an easy visualization of the data, which were then analyzed to determine whether motion direction and frequency affected the response independently of each other or whether an interaction between these stimulus dimensions became apparent.

These early applications of the iso-response paradigm, however, did not aim at detailed characterizations of the non-linearities involved in stimulus integration. This requires high-precision measurements of iso-response stimuli, despite the limited recording time in physiological experiments. A key development for providing the required efficiency in the assessment of iso-response stimuli has been the possibility to use closed-loop experiments, benefiting from the recent colossal advancements in computer hardware and software.

## Measuring Iso-response stimuli with closed-loop experiments

**Figure 2:**
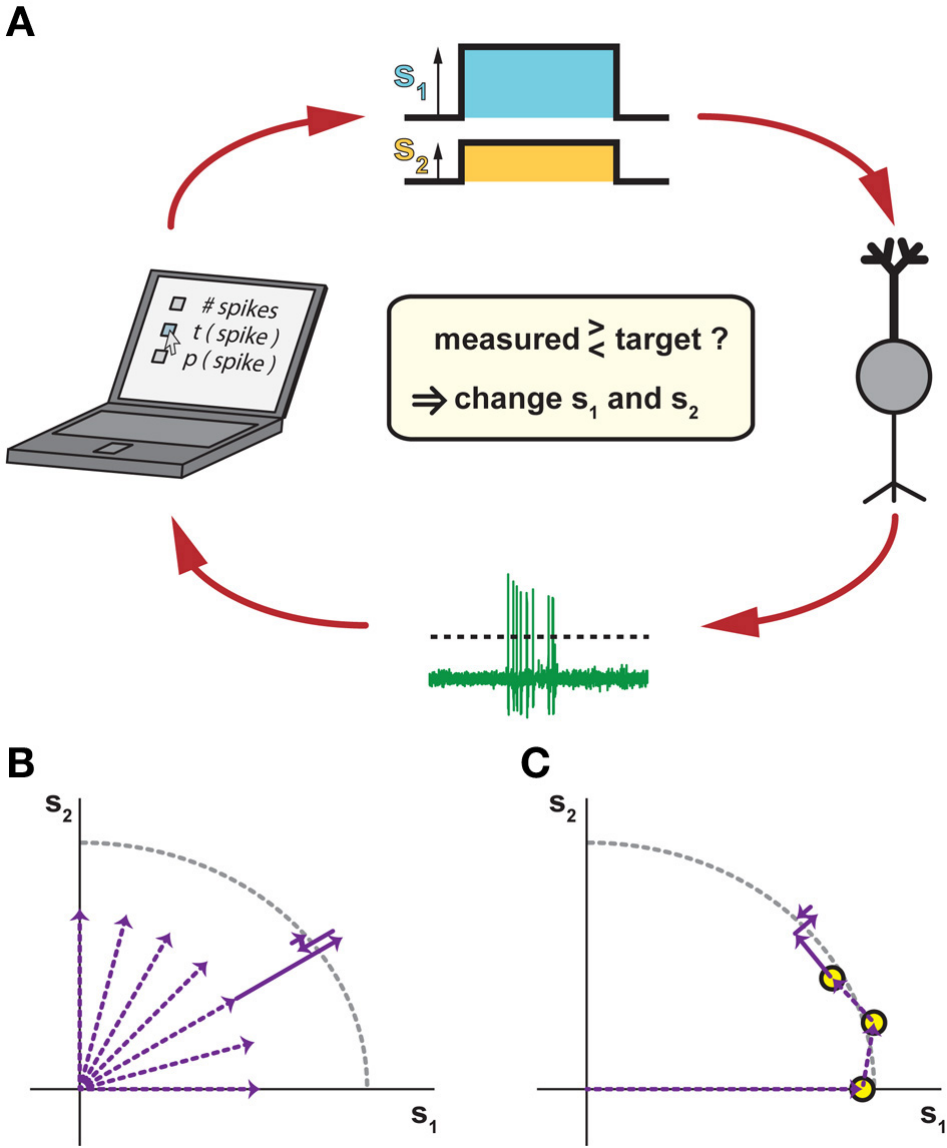
**Closed-loop methods for measuring iso-response stimuli. (A)** Closing the loop by tuning stimulus parameters according to measured responses. In response to a stimulus with two components s_1_ and s_2_ (top), a recorded neuron (right) responds with spikes that are automatically detected, for example by a threshold criterion (bottom). The spike response is then compared to a chosen target criterion (left), which may be the number of spikes, the timing of the first spike, the probability of spiking, or any other accessible response feature. According to this comparison, the values of s_1_ and s_2_ are adjusted for the next stimulus presentation in order to approach the target response. **(B)** Potential search strategy in radial directions of the stimulus space. This combines several linear searches, which can be performed sequentially or interleaved, typically starting near the origin so that overly strong stimulation is avoided. **(C)** Potential search strategy by tracking iso-response curves. Here, previously measured iso-response stimuli are used as starting conditions for searching nearby stimuli that yield the same response. This can be done, for example, by changing the ratio of s_1_ and s_2_ while keeping the same radial distance as for a previously measured iso-response stimulus and then tuning this radial distance until the desired response is obtained. As compared to the strategy of pure radial searches in **(B)**, this sequential search can provide higher recording efficiency, but does not allow interleaving multiple searches.

From an experimental viewpoint, the iso-response methodology suggests a conceptual change in the design of a neurophysiological experiment—instead of measuring how responses vary for different predefined stimuli, the goal is to manipulate stimuli such that the recorded cell's output stays at the same level, or at least remains within a small predefined range. This challenging task can only be accomplished efficiently within a closed-loop setting (Benda et al., 2007) so that information about changes in the neural output can immediately be fed back to the stimulus generator (Figure 2A).

In a first, exploratory phase of an iso-response experiment, the closed-loop setting is highly useful to determine which stimulus dimensions should be explored at all (e.g., which spatial locations or spectral components). In the second phase, the actual iso-response stimuli are determined. To do so, the closed-loop setup is used to implement a search algorithm. The search for a particular stimulus that provides a predefined response can, for example, proceed radially outwards from stimulus origin in different directions (Figure 2B). Alternatively, the search can move along an iso-response curve (Figure 2C) by starting at some stimulus and then searching in its vicinity for stimuli leading to the same response. The search for a stimulus that yields a predefined response is essentially a root-finding problem, for which many algorithms of varying efficiency and complexity exist (Press et al., 1992). Essentially, however, the search amounts to comparing the measured response to a target and deciding whether increasing or decreasing the strength of the stimulus components reduces the deviation. As it is not always possible to exactly reach the desired response value, the parameter values for these stimuli are often determined through interpolation from stimuli that led to responses within a small region around the set response. To save precious experimental time, this can also be done offline.

In either phase of the iso-response experiments, precise, flexible, and fast stimulus control is needed, as well as good control over the data acquisition, in particular regarding spike detection and spike sorting (Lewicki, 1998; Quiroga et al., 2004; Santhanam et al., 2004; Wood et al., 2004; Rutishauser et al., 2006). The rapid detection of iso-response stimuli through efficient closed-loop approaches can then not only be used to obtain high-accuracy measurements, but also allows one to measure and compare different variations of iso-response curves from the same cells. For example, it may help elucidate the mechanisms underlying the non-linearities of stimulus integration to repeat iso-response measurements in the presence of pharmacological blockers, for different response measures, or using different stimulus components as the inputs s_1_ and s_2_. To illustrate the power and potential of closed-loop methods for iso-response measurements, we will, in the following, summarize some key ideas and results of recent applications of this method in different sensory systems.

## Example I: the auditory periphery of locusts

We begin with the integration of acoustic stimuli in locust auditory receptor cells. In this model system, three different types of iso-response experiments have been performed to address several distinct questions. In a first study, iso-firing rate stimuli were used to discriminate between rival hypotheses for spectral integration of sound signals (Gollisch et al., 2002). In a second study, iso-spike probability experiments revealed temporal integration mechanisms on a sub-millisecond scale (Gollisch and Herz, 2005). In a third study, iso-firing rate stimuli were used once more, but they were now designed such that different adaptation mechanisms could be discerned (Gollisch and Herz, 2004). Together, the three iso-response studies led to new insights and quantitative results far beyond the scope of traditional experiments.

Locust auditory receptor neurons are directly attached to the animal's eardrum via short dendrites. When the eardrum vibrates in response to incident sound, mechanosensory ion channels in the neurons open (Gillespie and Walker, 2001). The transduction currents cause depolarizations of the neuronal membrane and thereby trigger spikes, which can be recorded from the receptors' axons in the auditory nerve (Hill, 1983a). Individual receptor cells are broadly tuned to sound frequencies above a few kilohertz and do not phase-lock to the sound's carrier frequency (Hill, 1983b).

Returning to the example from the introduction, let us consider sound pressure waves s(t) that consist of superimposed pure tones, s(t) = Σ_i_ s_i_ cos(2πν_i_t). How the cells' average firing rate *r* depends on sound intensity is subject to three rival hypotheses, in which *r* is considered to be a non-linear function *r* = *f*(*J*) of the “effective stimulus intensity” *J*, which in turn represents a different fundamental measure of sound intensity according to each hypothesis (Garner, 1947; Tougaard, 1996; Heil and Neubauer, 2001): *Amplitude Hypothesis*: *J* is proportional to a weighted sum of the tone amplitudes, *J*_AH_ = Σ_i_λ_i_ s_i_, where the factors λ_i_ represent the relative sensitivities of the eardrum to different sound frequencies. Thus, *J* reflects the maximum amplitude of the eardrum vibration. *Energy Hypothesis: J* corresponds to the energy of the eardrum oscillations, *J*_EH_ =Σ_i_λ^2^_i_ s^2^_i_. *Pressure Hypothesis: J* corresponds to the temporal mean of the absolute value of the oscillation, *J*_PH_ = <|ŝ(t)|>, where ŝ(t) describes the sound pressure wave after taking the sensitivities λ_i_ into account.

Which of these three hypotheses applies to locust auditory receptors? Answering this question about the true physical cause of output activity is complicated by the strongly non-linear dependence of *r* on *J* through the output non-linearity *f* and because *J* cannot be determined directly since the locust auditory system is very delicate so that one cannot reliably measure sound transduction prior to the receptor cells' spike generation. Thus, to investigate stimulus integration independently of *f*, the iso-response paradigm was implemented, using superpositions of two sine-wave stimuli in order to identify those amplitude combinations that led to the same firing rate. Note that for each of the three hypotheses, the stimulus-response relation takes on the form of the canonical model of Figure 1A: the output non-linearity N_2_ is always given by the function *f*, whereas N_1_ is either just a linear function (amplitude hypothesis), a squaring relation (energy hypothesis), or a more complicated non-linearity that has to be determined numerically (pressure hypothesis). The iso-response curves can thus distinguish between the three hypotheses independently of the non-linear relation between the effective stimulus strength and the firing rate.

**Figure 3:**
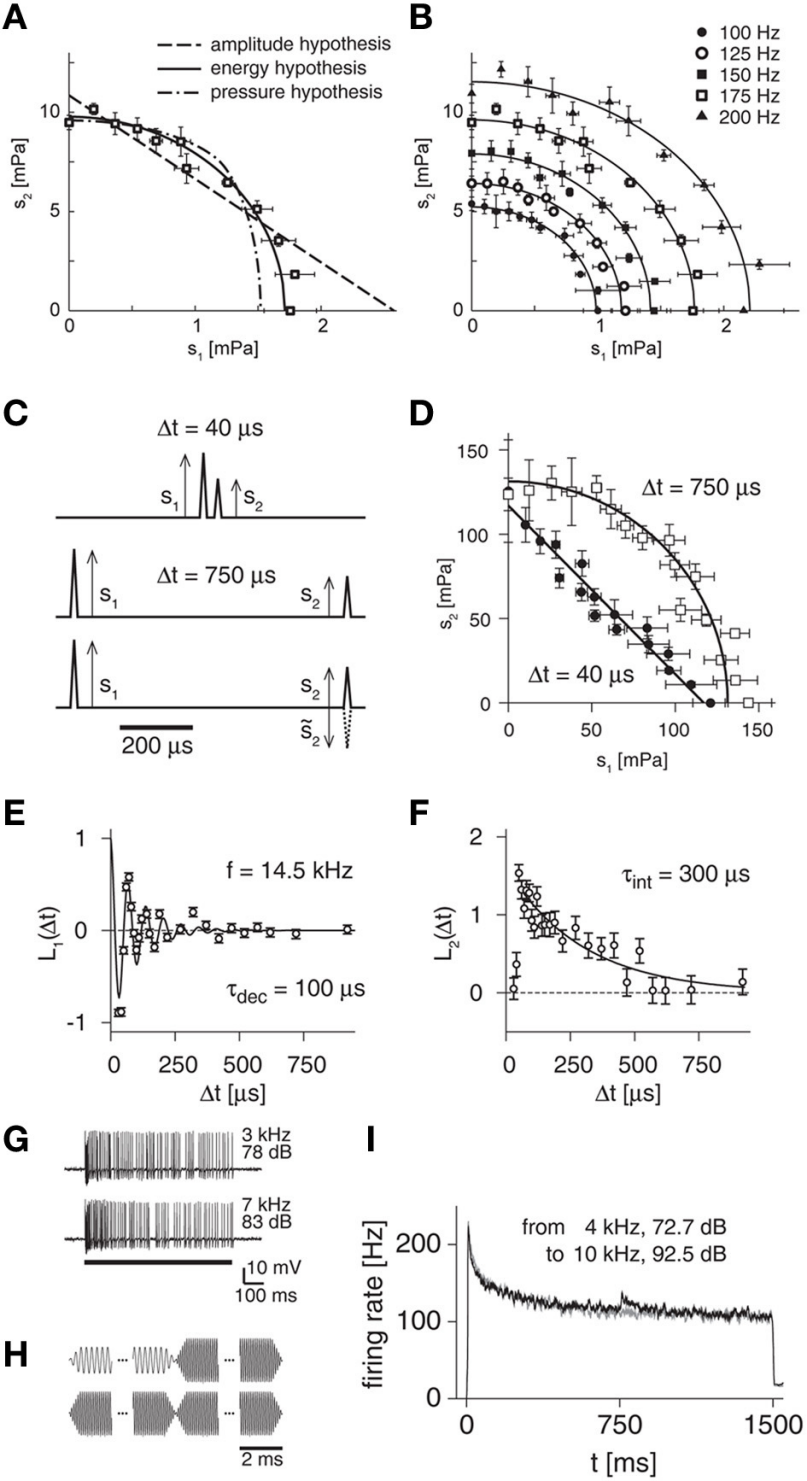
**Iso-response measurements for locust auditory receptors. (A)** Iso-response curve of spectral integration to distinguish between three rival hypotheses of sound transduction. Stimuli consisted of superpositions of two pure tones with amplitudes s_1_ and s_2_, respectively. The data points show such combinations that yielded a firing rate of 175 Hz. These are well fitted by an ellipse, corresponding to the energy hypothesis, but not by iso-response curves of the other two model hypotheses. **(B)** Comparison of iso-response curves from the same cell as in **(A)** for different target response levels. The iso-response curves are ellipses that are scaled versions of each other, confirming the energy hypothesis. **(C)** Click stimuli used to probe temporal integration. The clicks can be presented with short (top) or long (middle) inter-click intervals, and the second click can have the same or opposite sign as compared to the first (bottom). **(D)** Iso-response curves for pairs of clicks at different inter-click intervals, corresponding to a spike probability of 70%. Short inter-click intervals yield straight lines, longer inter-click intervals result in circles, corresponding to linear and quadratic temporal integration, respectively. **(E)** Linear filter L_1_ for a sample receptor cell obtained from iso-response measurements with different signs of the second click. The filter corresponds to the first stage of the LNLN model of sound transduction (Figure 1D). The filter shape can be fitted by an impulse response function of a damped oscillator (black line) with oscillation frequency *f* and (mechanical) decay time constant τ_dec_ as indicated in the plot. **(F)** Linear filter L_2_ for the same receptor cell as in **(E)**. The filter shape can be fitted by an exponential function (black line) with (electrical) integration time constant τ_int_ as indicated in the plot. **(G)** Sample spike trains recorded from a locust auditory receptor neuron for presentations of pure tones, showing the adaptation of the firing rate during the course of stimulation. **(H)** Acoustic stimuli used to test for input-driven adaptation. Amplitudes of a high-frequency tone and a low-frequency tone were tuned with closed-loop experiments so that they evoked the same steady state firing rates. Stimuli then consisted of switches between the tones (top) as well as of repetitions of the same tone as control condition (bottom). **(I)** Firing-rate profile for a sample receptor neuron. For the switch from one tone to another, the firing rate displays a transient increase (black line), which is absent in the control condition (gray line). This indicates that besides the strong feedback adaptation driven by the neuron's spikes, an additional adaptation component exists before signals are integrated over sound frequencies. Panels **(A)** and **(B)** reprinted from Gollisch et al. (2002) with permission, Copyright (2002), *Journal of Neuroscience*, Society for Neuroscience. Panels **(C–F)** adapted from Gollisch and Herz (2005) according to the Creative Commons Attribution License. Panels **(G–I)** reprinted from Gollisch and Herz (2004) with permission, Copyright (2004), *Journal of Neuroscience*, Society for Neuroscience.

As indicated in Figure 3A for an exemplary receptor neuron, the amplitude and pressure hypotheses were rejected by the measured shapes of iso-response curves, whereas the energy hypothesis provided a good fit to the data (Gollisch et al., 2002). To test the generality of this conclusion, a useful extension is to investigate how iso-response curves for different response levels are related to one another (Figure 3B). For locust auditory receptor neurons, iso-firing rate curves obtained for the same neuron at different firing rates turned out to lie on ellipses that are scaled versions of one another. Again, this finding is in accordance with the energy hypothesis, which predicts that the ratio of the ellipses' half-axes should always equal the ratio of the constants λ_1_ and λ_2_. In addition, the energy model also holds for the initial transient response at stimulus onset as well as for superpositions of multiple pure tones and even accurately predicts receptor responses to broad-band noise stimulation (Gollisch et al., 2002).

These observations led to the conclusion that sound-intensity coding in this insect model system is well captured by a cascade model (Figure 1D), in which the sound wave is first mechanically filtered by the eardrum and the transduction stage then provides a squaring non-linearity prior to temporal integration of the electrical signals in the receptor neuron. A non-linear output stage finally describes the firing-rate encoding of the effective sound intensity *J*_EH_, resulting in an LNLN-cascade. The temporal dynamics of this cascade, in particular of the different filtering stages, however, were beyond the reach of this first set of experiments with stationary stimuli.

Instead, disentangling the characteristics of temporal integration in sound encoding requires the application of highly dynamic stimuli. Accordingly, the iso-response paradigm was extended to a response measure appropriate for such a dynamic scenario, namely the probability of occurrence of a single spike following a brief stimulus (Gollisch and Herz, 2005). Thus, iso-response curves were measured for double-click stimuli with inter-click intervals of less than one millisecond. The two click amplitudes s_1_ and s_2_ were again adjusted via a closed-loop search algorithm during an experiment such that a recorded cell responded to repeated stimulation with a fixed spike probability.

When the inter-click intervals were sufficiently large (Figure 3C, middle panel), the iso-response curves were approximately circular (Figure 3D, open squares). This finding further corroborates the energy hypothesis, as the circular iso-response curve shows that equal spike probability was obtained for equal sound energy, s^2^_1_ + s^2^_2_. When very short inter-click intervals were chosen (Figure 3C, top panel), however, the iso-response curves were nearly straight lines (Figure 3D, filled circles). Thus, on short times scales, the sum of the two click amplitudes, s_1_ + s_2_, determines the spike probability. This is readily explained if one assumes that the two stimulus components are already mechanically integrated by the oscillation of the eardrum, which is expected to act as a linear filter for the sound-pressure wave (Schiolten et al., 1981).

The different shapes of the iso-response curves on different time scales imply that different integrative steps are relevant during the mechanosensory transduction process. This is expected, as the sound pressure wave is first mechanically filtered by the eardrum. After conversion into electrical signals, these are integrated by the capacitive properties of the neuron's cell membrane. In the LNLN cascade of sound transduction (Figure 1D), the two temporal integration steps are captured by the linear filters L_1_ and L_2_, respectively. How can the temporal structure of these two filters, separated by the squaring non-linearity of mechanosensory transduction, be disentangled? The solution again lies in properly designed iso-response measurements, here by comparing the click amplitudes necessary to evoke the same spike probability when the pressure deflection of the second click either has the same or the opposite sign of the pressure deflection of the first click (Figure 3C, bottom panel). The rationale behind this approach is that the linear integration before the squaring non-linearity is sensitive to a change in sign, whereas the integration following the squaring transformation is not. Using the mathematical description of the LNLN cascade, this reasoning can be cast into formulas for extracting filter shapes of L_1_ and L_2_ at different time points, which correspond to the applied inter-click intervals (Gollisch and Herz, 2005).

This approach showed that L_1_ resembles the filter of a damped oscillator (Figure 3E). In fact, the measured resonance frequencies of these oscillators corresponded to the receptor cells' maximal spectral sensitivity, which typically lies in the range of several kilohertz (Gollisch and Herz, 2005). In addition, these measurements revealed damping time constants of typically few hundred microseconds, thus providing insight into the mechanical eardrum properties at the different sites where the receptor cells are attached. By contrast, the second filter L_2_ rather had the shape of a leaky integrator with exponential decay characteristics, thus showing the time scales of electrical integration at the cell membrane (Figure 3F). Typically, the decay of L_2_ was slower than that of L_1_. Thus, long inter-click intervals surpass the mechanical integration and rather reveal the quadratic integration characteristics of electrical signals as evident in the approximately circular iso-response curves for sufficiently long inter-click intervals (Figure 3D).

Note that the assessment of the integration dynamics on time scales as short as few tens of microseconds could be achieved by measuring the spike probability with comparatively large temporal windows of several milliseconds. This makes the approach insensitive to variability in spike timing, which mars the temporal resolution of traditional correlations techniques (Aldworth et al., 2005; Dimitrov and Gedeon, 2006; Gollisch, 2006). By contrast, the temporal resolution in these iso-response measurements is limited only by the accuracy of stimulus delivery, which may easily reach the microsecond range with appropriate hardware and software.

On much longer time scales, many neurons exhibit spike-frequency adaptation (Figure 3G). An initially high firing rate slowly decreases over time, even though the stimulus stays constant. There is a wide range of different biophysical mechanisms known to be involved in spike-frequency adaptation. In many neurons, a major contribution stems from output-driven components that are triggered by the spiking activity of the neuron. Adaptation may, however, also contain components that are driven by the sensory or synaptic input in a feed-forward way. The different dependences of adaptation on the sensory input and neural output will have distinct effects on the coding properties of a sensory neuron. For a functional characterization of adaptation, we therefore have to identify the causal relationships between sensory input, neural activity, and the level of adaptation.

To tackle this problem, one needs to measure input-driven adaptation, which is triggered by the strength of a stimulus component s_i_, independently of output-driven adaptation, which follows the total response level of the neuron. Applying again the iso-response approach to auditory receptor neurons, this can be done by tuning the intensities for different sound frequencies in such a way that the steady-state firing rate is the same (Gollisch and Herz, 2004). Consequently, the level of output-driven adaptation must be equal. Switching between these sounds (Figure 3H) can then reveal input-driven components, because these need to approach a new equilibrium value after such a switch. This process results in transient deflections of the firing rate, which can be observed in electrophysiological recordings of the spiking activity (Figure 3I). The careful tuning of the sounds leads to a high sensitivity of the method that allows one to detect input-driven adaptation components even when they are far smaller in effect than simultaneously present output-driven components.

## Example II: retina

The vertebrate retina is a neural network at the back of the eyeball that constitutes the first stage of visual processing. The processed visual signals are encoded by retinal ganglion cells into patterns of spikes for transmission along the optic nerve to various brain regions. As in many other sensory systems, the network of the retina features a great deal of convergence; a single ganglion cell can collect signals from tens to hundreds of excitatory bipolar cells (Freed and Sterling, 1988), which in turn each collect signals from many photoreceptors. Inhibitory interactions mediated by horizontal cells and amacrine cells influence which signals are transmitted in this processing chain and how they are modified.

The spikes from an individual retinal ganglion cell thus reflect the processing of this complex upstream circuit. What the circuit computes follows to a large degree from the nature of the non-linearities associated with the ganglion cell's integration over its collection of inputs (Gollisch and Meister, 2010). That this integration can occur in a non-linear fashion has been known for more than fifty years, since ganglion cells were first categorized as linear X cells and non-linear Y cells (Enroth-Cugell and Robson, 1966). Yet, the classical experiments for identifying non-linear stimulus integration with reversing spatial gratings only indicate whether or not a non-linearity is present and do not directly reveal its functional form. Moreover, it is likely that the class of non-linearly integrating cells is composed of various types of ganglion cells, which may express different types of non-linear characteristics, serving different visual functions.

**Figure 4:**
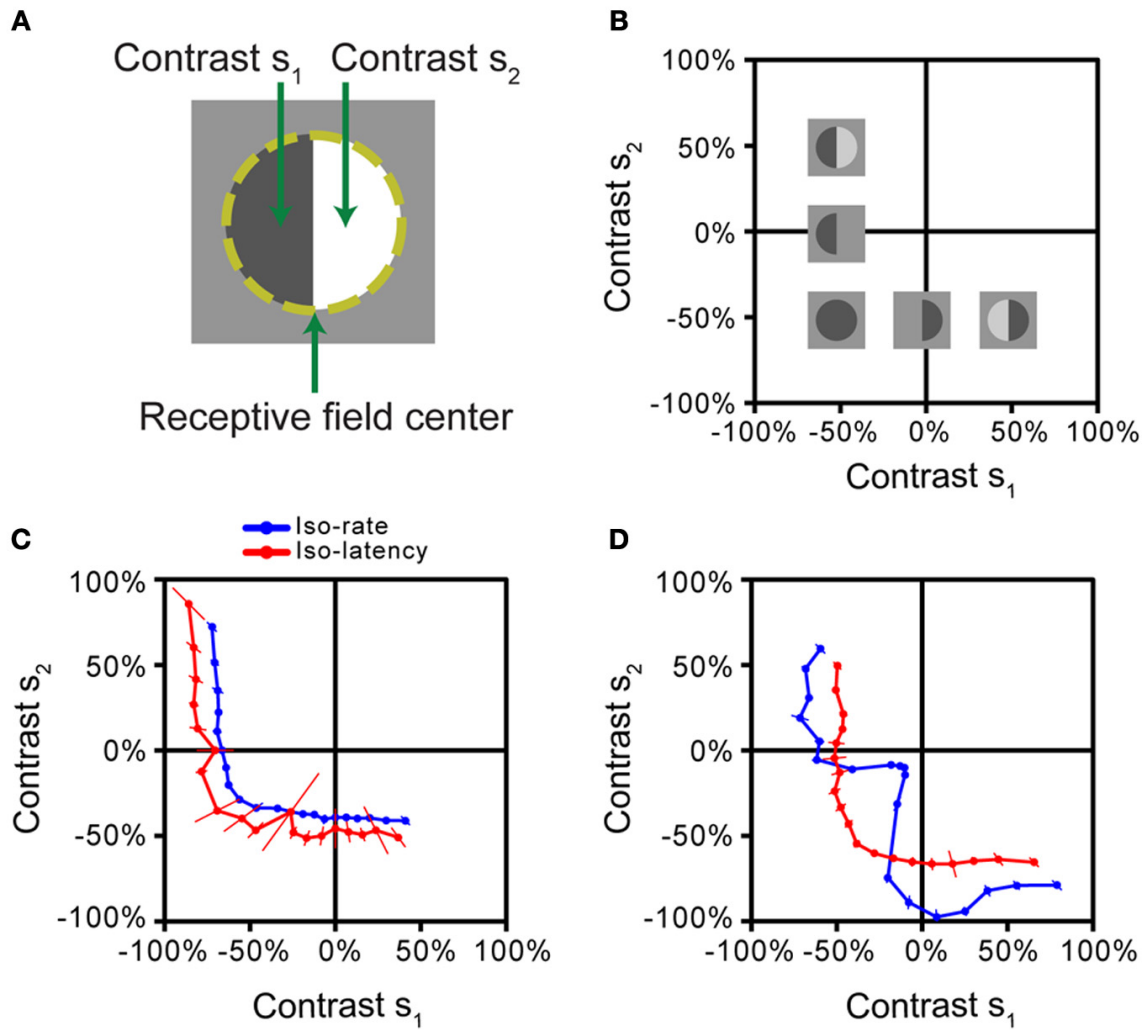
**Iso-response measurements of spatial stimulus integration by retinal ganglion cells. (A)** Stimulus pattern used in the measurements. After determining the receptive field center of a retinal ganglion cell (dashed line), different contrast levels s_1_ and s_2_ were simultaneously displayed for 500 ms, each in one half of the receptive field. **(B)** Stimulus space. Iso-response stimuli were measured in the space spanned by s_1_ and s_2_. Experiments were performed on Off-type ganglion cells, which best respond to negative contrast. Several sample stimulus patterns are shown at their respective locations in stimulus space. The origin corresponds to the gray level of background illumination. **(C)** Iso-rate and iso-latency curves for a sample ganglion cell, corresponding to the majority of recorded cells in the salamander retina. Both iso-response curves have similar shapes that indicate a threshold-quadratic non-linearity of stimulus integration. **(D)** Iso-rate and iso-latency curves for a different ganglion cell from a subpopulation in the salamander retina. While the iso-latency curve has a similar shape as the curves in **(C)**, the iso-rate curve shows a notch along the lower-left diagonal, corresponding to particular sensitivity to homogeneous stimulation of the receptive field. This follows from a dynamic local gain control mechanism, mediated by inhibitory interactions. All panels reprinted from Bölinger and Gollisch (2012), Copyright (2012), with permission from Elsevier.

Based on the iso-response paradigm, the nature of stimulus integration in the receptive field can be analyzed by subdividing the receptive field into two halves (Figure 4A) and using the values of the visual contrast in each half as inputs, analogous to the canonical model of Figure 1A. This approach has recently been applied to measuring stimulus integration by Off-type ganglion cells in the salamander retina (Bölinger and Gollisch, 2012). The contrast combinations (s_1_, s_2_) were flashed briefly onto the receptive field of a ganglion cell, whose spikes were recorded extracellularly. Closed-loop experiments were then used to find such combinations that either gave the same spike count (iso-rate curves) or the same first-spike latency (iso-latency curves). As the stimulus started from an intermediate gray illumination, both positive contrast (brightening) as well as negative contrast (dimming) could be applied, and iso-response stimuli were therefore measured beyond just one quadrant of stimulus space (Figure 4B).

The iso-response curves revealed that all measured ganglion cells in the salamander retina featured non-linear stimulus integration. For the majority of cells, iso-rate curves and iso-latency curves had the same general shape, as shown by an example in Figure 4C. The curves were approximately circular in the region where both contrast values were negative (corresponding to the preferred contrast for these Off-type cells). In this region, the curves thus resembled the circular iso-response curves seen in a simple model (Figure 1C) and in the previous example (Figure 3A), suggesting that a sum of squares determines the response of these ganglion cells. Combinations of negative contrast in one half of the receptive field and positive contrast in the other, however, yielded sections of the iso-response curves that were nearly parallel to the axes of the plot. This suggests that the amount of positive contrast had little or no effect on the response strength, corresponding to a thresholding mechanism that implements a half-wave rectification. Together, the shape of these iso-response curves indicates that a threshold-quadratic transformation is the fundamental non-linearity of stimulus integration over the receptive field center of these ganglion cells.

Other recorded ganglion cells, however, showed a fundamentally different shape of the iso-rate curves (Figure 4D). Instead of the circular shape in the region where both contrast values are negative, the curves show a pronounced notch, indicating that particularly small contrast levels were required to reach the target spike count when both receptive field halves were stimulated with the same negative contrast. Accordingly, the cells were named “homogeneity detectors,” as they appear particularly suited to detect large, homogeneous objects, even at low contrast (Bölinger and Gollisch, 2012).

Both types of ganglion cells, those with threshold-quadratic non-linearities as well as homogeneity detectors, are strongly non-linear in their integration characteristics. They would thus both be classified as Y-type cells according to a conventional investigation of linear vs. non-linear stimulus integration with reversing grating stimuli (Enroth-Cugell and Robson, 1966; Bölinger and Gollisch, 2012). The assessment of integration characteristics with iso-response curves, on the other hand, allowed an analysis of the particular type of non-linearity in a quantitative and detailed fashion and thus provided a distinction between different types of non-linear stimulus integration that had not been apparent before.

Interestingly, the iso-latency curves of homogeneity detectors did not display the characteristic notch, but rather showed the circular region, similar to the majority of measured iso-rate curves. The comparison between iso-rate and iso-latency curves thus already provides insights regarding the mechanism responsible for the characteristics of homogeneity detectors; it suggests that sensitivity to homogeneous stimuli is obtained through a process that acts only after the first spike is initiated and thus has a dynamic nature. Further investigations showed that this phenomenon is brought about by local inhibitory circuitry, acting as a local gain control and coming into effect with a slight delay because of the additional synaptic stage involved in the inhibitory pathway (Bölinger and Gollisch, 2012).

## Example III: visual cortex

**Figure 5:**
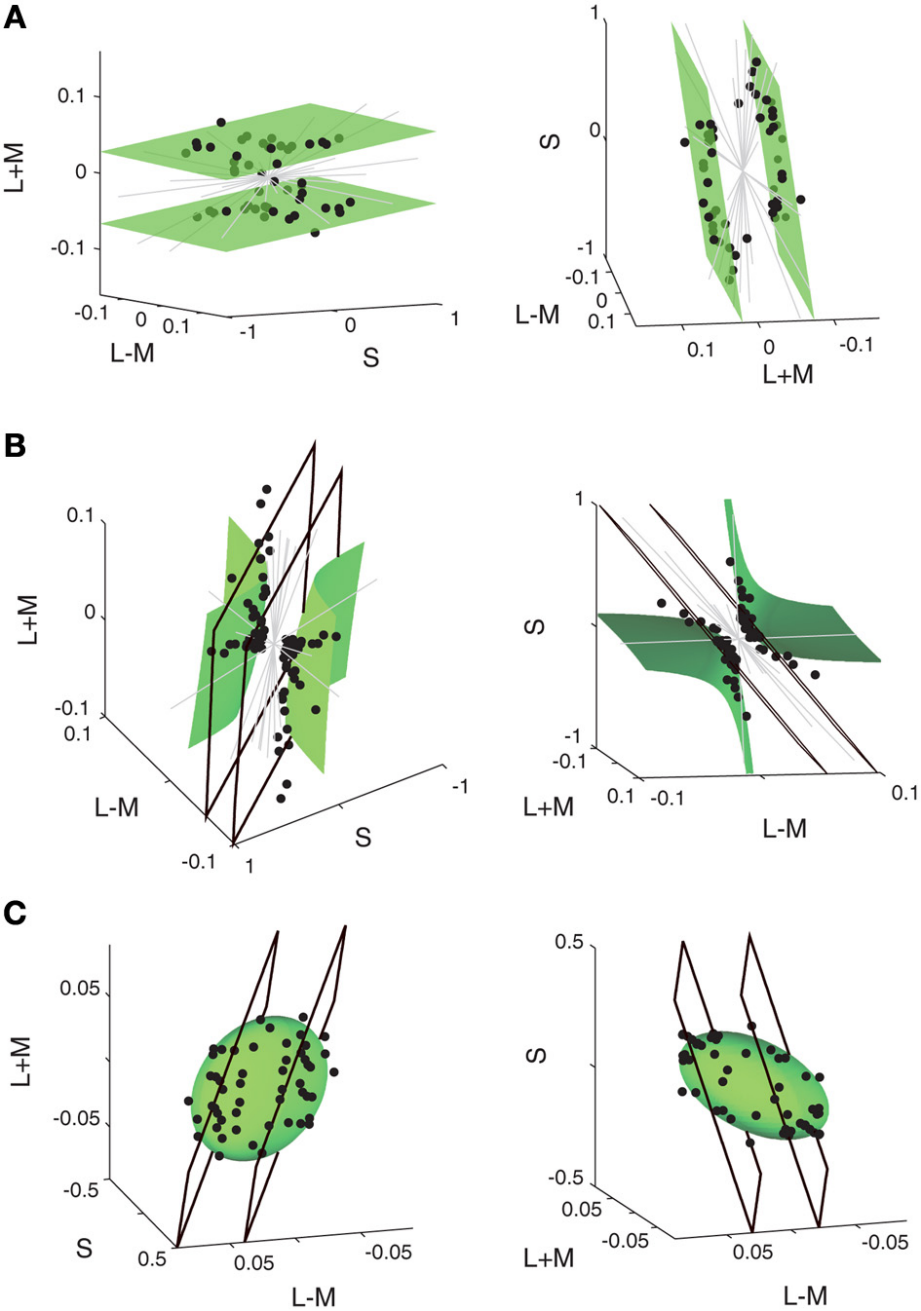
**Iso-response measurements of integration of cone signals by neurons in macaque primary visual cortex.** The panels show iso-response stimuli (black circles) obtained with drifting chromatic gratings for three representative sample cells in **(A)**, **(B)**, and **(C)**, respectively. Stimuli that yielded the same firing rate are plotted in the space spanned by S-cone activation (S) and by the sum and difference of L-cone and M-cone activation (L + M and L − M, respectively). For each cell, the data are shown in the 3D plots for two different viewpoints (left and right column, respectively). Gray lines indicate directions in stimulus space along which the predefined response criterion could not be reached. As shown by the green surface plots, iso-response stimuli are well fitted by a linear plane for the cell in **(A)**, by a hyperboloid for the cell in **(B)**, and by an ellipsoid for the cell in **(C)**. Panels **(B)** and **(C)** also show best fits of linear planes (black quadrangles), which do not provide good descriptions of the iso-response stimuli. Reprinted by permission from Macmillan Publishers Ltd: Nature Neuroscience (Horwitz and Hass, 2012), copyright (2012).

A further recent application of iso-response measurements has shed light onto the integration of color information by neurons in primate visual cortex (Horwitz and Hass, 2012). This study was motivated by the puzzle that neuronal responses in visual cortex to color stimuli often appeared incongruent with representing linear sums and differences of cone signals, an expectation that had been developed on the basis of psychophysical color perception experiments (Hering, 1920; Hurvich and Jameson, 1957). To resolve this issue and test whether non-linear integration of cone signals had been a missing ingredient in the models with which the data had been analyzed, Horwitz and Hass (2012) measured iso-response surfaces of macaque V1 neurons in a three-dimensional color stimulus space, defined by the activation of the three types of cones in the retina (Figure 5). Using drifting chromatic gratings as stimuli, the iso-response stimuli were defined as those combinations of cone activation that elicited the same firing rate over the stimulus duration.

The iso-response stimuli define two-dimensional surfaces in this three-dimensional stimulus space. For some cells, the iso-response surfaces were simple planes (Figure 5A), indicating that these cells represent indeed a linear combination of cone activation strengths. Other cells, however, showed strong deviations from linear integration; for those cells, the iso-response data points were much better fitted by quadratic models, either corresponding to a hyperboloid (Figure 5B) or to an ellipsoid (Figure 5C). Taken together, the data show that iso-response surfaces of individual cells are generally well described by either a linear or a quadratic integration model. This finding demonstrates that the previous lack of a coherent description of cortical responses to color stimuli in terms of cone activations resulted from not taking non-linear integration into account.

Interestingly, the hyperboloid iso-response surface of Figure 5B is similarly non-convex as the iso-response curve of homogeneity detectors measured in the retina (Figure 4D). This shape suggests that the cells are especially sensitive to one particular stimulus dimension—homogeneous stimulation of the receptive field in the case of the retinal neuron; a particular cone activation pattern in the case of the cortical neuron—whereas responses in other directions appear suppressed; in the case of the cortical neuron, this means that for certain combinations of cone activation, the desired response is never reached. One may thus hypothesize that the hyperboloid shape of the iso-response surface in cortical neurons is brought about by a similar active suppression mechanism as mediated by local inhibition in the case of the retinal homogeneity detectors.

The iso-response surface in the shape of an ellipsoid (Figure 5C), on the other hand, indicates that the cell represents a sum of squares, similar to findings in both the locust auditory system (Figure 3A) and the salamander retina (Figure 4C) as well as in the energy model for complex cells in visual cortex (Adelson and Bergen, 1985). The ubiquity of this type of non-linear stimulus integration may indicate a general-purpose representation, providing invariance under rotations in stimulus space.

## Multiple stages of stimulus integration

The canonical model of Figure 1A suggests that the iso-response method is most easily applied to systems with two non-linear stages, one before stimulus integration has taken place and one afterwards. Yet, valuable insight can also be obtained for systems with more successive non-linearities. First, from a functional point of view it may not be necessary to disentangle all non-linear stages; rather, it may be of interest to determine the total, combined non-linear transformation before stimulus integration takes place and separate it from the total non-linearity afterwards. This procedure aims at casting the investigated system again into the form of the canonical NLN cascade of Figure 1A, but will fail for systems that deviate strongly from this simplified structure.

Second, one may profit from the fact that many neural systems, in particular sensory systems, are organized in a hierarchical fashion so that the relevant temporal, spatial, and spectral scales increase from processing layer to processing layer. This allows one to choose the stimulus layout—by appropriately defining what is represented by the two components s_1_ and s_2_—in such a way that the relevant stimulus integration occurs at a certain stage along the processing chain, dividing the chain into the total non-linear transformation before and after this stage. By varying the stimulus scale used in the analysis, one can thus distinguish between successive non-linear stages.

**Figure 6:**
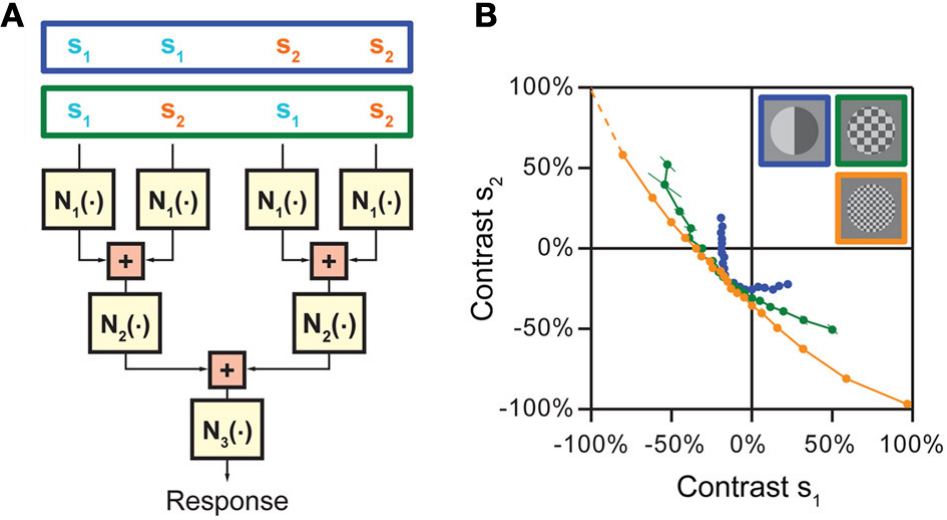
**Approach for disentangling non-linearities at multiple stages of stimulus integration in hierarchical models. (A)** Cascade model with three consecutive non-linear stages, N_1_, N_2_, and N_3_, separated by two integration stages. The model assumes that first nearby stimulus components are integrated, whose results are then combined in a subsequent stage. Different stimulation schemes that can be used to separate the effects of the non-linearities are shown on top. When nearby input channels are stimulated with the same stimulus component s_1_ or s_2_, respectively (stimulus pattern in blue box), the iso-response curve is affected by the combination of N_1_ and N_2_. When the two stimulus components s_1_ and s_2_ are placed so that they are combined already by the first integration stage (stimulus pattern in green box), only non-linearity N_1_ is relevant for the shape of the iso-response curve. **(B)** Application of the strategy to separate different integration stages of spatial integration by retinal ganglion cells. When nearby spatial locations receive the same contrast (blue data points), the iso-rate curve shows the standard threshold-quadratic non-linearity as in Figure 4C. When the two contrast components s_1_ and s_2_ are interleaved so that presynaptic bipolar cells typically already start integrating the two components, but individual photoreceptors only receive either one of the components (green and orange data points, corresponding to squares in the stimulus layout with 150 and 60 μm side length, respectively), the iso-rate curves approach straight lines, showing that the integration stage from photoreceptors to bipolar cells can be approximated as linear integration. Panel **(B)** reprinted from Bölinger and Gollisch (2012), Copyright (2012), with permission from Elsevier.

To illustrate this strategy, let us consider a model with three non-linear stages N_1_, N_2_, and N_3_, separated by successive stages of stimulus integration, which first only pool over sets of neighboring inputs and subsequently integrate over these sets (Figure 6A). To separate these integration stages, we now first choose a “coarse” stimulus layout, in which the four input channels are combined into pairs so that “nearby” channels, which are pooled together already in the first integration stage, receive the same stimulus intensity s_1_ or s_2_, respectively (stimulus pattern inside the blue box in Figure 6A). For this stimulus layout, s_1_ and s_2_ remain separate through both N_1_ and N_2_ and are combined only prior to the output non-linearity N_3_. This means that the iso-response curve of s_1_ and s_2_ will reflect the concatenation of N_1_ and N_2_, but is not influenced by N_3_. Now, let us consider a “finer” stimulus layout, in which “nearby” input channels already receive different stimulus components s_1_ and s_2_ (stimulus pattern inside the green box in Figure 6A). For this layout, s_1_ and s_2_ are combined directly after N_1_ and before N_2_, which means that the iso-response curve of s_1_ and s_2_ will now only reflect the non-linearity N_1_ and be insensitive to both N_2_ and N_3_. Investigating and comparing the shapes of iso-response curves on a fine and coarse scale thus can be used to derive both non-linearities N_1_ and N_2_. Finally, for completeness, N_3_ could simply be obtained by homogeneously stimulating all four input channels with the same, varying stimulus intensity, thus measuring the combined effect of all three non-linear stages, and comparing this to the effect of N_1_ and N_2_ alone.

The strategy of comparing iso-response curves measured with coarse and fine stimulus layouts has been used to track the origin of the non-linearities in the receptive fields of retinal ganglion cells that were described in Figure 4 (Bölinger and Gollisch, 2012). Spatial stimulus integration in the retina occurs successively from photoreceptor cells via biopolar cells to ganglion cells. These integration stages cover different spatial scales; photoreceptor cells integrate light over a distance of about 10 μm (Mariani, 1986; Sherry et al., 1998), whereas bipolar cells have receptive fields of roughly 50–100 μm diameter (Wu et al., 2000; Baccus et al., 2008) and ganglion cells in the range of 200–600 μm. Thus, analyzing whether the non-linear structures of iso-response curves persist or change on spatial scales below several tens of micrometers allows one to test whether the site of the non-linearity is before or after stimulus integration by bipolar cells. This concept has been applied by arranging the stimulus components in a checkerboard-like fashion with different sizes of the individual checkerboard fields. Measurements of iso-response stimuli then showed that, as the scale of the fields fell roughly below 100 μm, the shapes of iso-response curves approached straight lines (Figure 6B). This meant that no relevant non-linearity occurred between photoreceptor cells and bipolar cells; to good approximation, stimuli were integrated linearly by bipolar cells.

Essentially the same principle was also behind the separation of different integration stages in locust auditory receptor neurons, as discussed above, by probing the system with pairs of acoustic clicks at different inter-click intervals (Figure 3C; Gollisch and Herz, 2005). For very short inter-click intervals, iso-response curves showed linear integration of the two clicks, corresponding to the linear mechanical integration at the eardrum; for longer inter-click intervals that surpassed the mechanical integration time, the quadratic non-linearity of transduction became apparent (Figure 3D).

## Comparison with spike-triggered covariance analysis

The iso-response method aims at identifying non-linear interactions in consecutive stages of neuronal processing. This relates the method conceptually to cascade models and reverse-correlation techniques, such as STA and STC analysis. As already discussed above, STA analysis fails to capture non-linear integration, because all stimulus integration is assumed to occur linearly in the single-filter LN model. STC analysis and related information-theoretic techniques (Paninski, 2003; Sharpee et al., 2004; Pillow and Simoncelli, 2006), on the other hand, provide multiple filters and a corresponding multi-dimensional non-linearity. While the popularity of STC analysis primarily rests on its ability to determine the number and shapes of relevant filters, it also, in principle, allows studying non-linear stimulus integration by analyzing the features of the multi-dimensional non-linearity. A primary challenge for this is again the need to separate non-linearities of stimulus integration from the non-linearity at the output stage. If no explicit models of the output non-linearity are available, calculating iso-response curves within the multi-dimensional stimulus subspace that is spanned by the identified filters (Rust et al., 2005) appears to be the method of choice for identifying non-linearities of stimulus integration, even if these iso-response curves must be computed in an offline fashion.

Note, however, that there are important practical differences between analyzing non-linear stimulus integration with STC analysis or with closed-loop iso-response measurements. STC analysis is based on continuous, stationary stimulation, typically with white-noise statistics. The closed-loop iso-response method, on the other hand, can also be applied under non-stationary presentation of individually analyzed stimulus segments and can thus be used also for fairly brief stimuli, such as flashed visual images or short sound bursts. This difference in stimulus statistics can have interesting consequences for the processing features of the investigated system. For example, high-threshold inhibition from amacrine cells in the retina (Bölinger and Gollisch, 2012) may be effectively absent in white-noise experiments, but contribute to ganglion cell processing for flashed or saccade-like image presentations.

Second, STC analysis can yield a fairly large number of filters, and the high dimensionality of the associated stimulus subspace may impede a detailed analysis of the non-linear stage (Rust et al., 2005). Unless spiking is well described by a Poisson process, the temporal dynamics of spike generation alone can lead to a collection of several relevant filters (Agüera y Arcas and Fairhall, 2003; Agüera y Arcas et al., 2003). Along the same line, STC analysis of retinal ganglion cells with purely temporal stimuli has been shown to yield multiple temporal filter components (Fairhall et al., 2006). When on top of temporal variations, stimuli have further structure, such as spatial dimensions, one obtains additional filters, including filter combinations that mix temporal effects with other stimulus dimensions. A detailed analysis of the full non-linear stage then easily becomes impractical, in particular for more than two or three dimensions, both for reasons of graphical display and required amounts of data. As a feasible alternative, one may aim at analyzing non-linearities in low-dimensional subspaces, for example, spanned by just two selected filters (Rust et al., 2005). However, all other relevant filters then effectively act as noise sources, reducing the efficiency of this analysis. The closed-loop iso-response approach circumvents this problem by focusing on a chosen, small-dimensional set of stimulus components, such as two spectral or spatial stimulus components. This becomes particularly useful when combined with prior closed-loop identification of appropriate stimulus components, for example, by matching the components to the location and size of a receptive field. The possibility to focus on few purposefully selected stimulus components as well as on a narrow response regime is the benefit of the technically more demanding closed-loop approach. Yet, the selected components remain a choice of the experimenter under the assumption that these correspond to meaningful, separate input channels for the neuron under study.

In this view, STC analysis and iso-response measurements are complementary. While the strength of the STC analysis lies mostly in determining—with relatively few prior assumptions—the number and nature of stimulus features that are non-linearly integrated, the iso-response method assumes certain stimulus components to be relevant features and aims at determining their non-linear integration in detail. For systems with little prior expectation about the relevant input channels, it may well make sense to base a closed-loop measurement of iso-response stimuli on the results of a prior STC analysis for guiding the choice of the applied stimulus components.

## Next steps and future challenges

As shown by the above examples, the iso-response method provides a powerful concept for studying how neurons integrate sensory inputs. Using different types of stimuli allows one to focus on spectral, spatial, temporal, or spatio-temporal integration. Exploring and comparing different output measures, such as firing-rate or first-spike latency, provides valuable insight into potential coding schemes. Furthermore, unlike correlation-based approaches, the temporal resolution of the iso-response method is *not* limited by the precision with which the output signal can be measured. This is best illustrated by the experiments where click-stimuli were presented to auditory receptor neurons whose output was measured in terms of the probability that a single, isolated spike is generated within a window stretching several milliseconds (Gollisch and Herz, 2005). The temporal filters L_1_ and L_2_ of the corresponding LNLN cascade were determined at a temporal resolution below 20 microseconds, restricted only by the precision of the acoustic stimulus generator. The stochastic nature of neural responses did not cause any limitations—in fact, the iso-spike-probability paradigm is only feasible because of a nonzero intrinsic noise level so that a single spike is generated in some, but not all trials. The critical, beneficial role of a neural characteristic that is usually considered an experimental nuisance was an interesting observation in these studies. In addition, one may think that the iso-response paradigm applies to conventional feedforward chains only; but as demonstrated by the study on input- vs. output-driven adaptation, certain feedback loops can also be studied with iso-response methods (Gollisch and Herz, 2004). We are thus confident that the iso-response paradigm will see further conceptual and methodological extensions in the future.

On the practical side, ongoing advances in soft- and hard-ware technology will increase the closed-loop interaction speed and also make it possible to include second-level analyses into the very design of iso-response experiments. This concern, for example, automated stopping rules in the search algorithms and automated selection of search directions, two developments of key importance for extending the iso-response approach to higher-dimensional search spaces. Closed-loop experiments have already been used to determine stimulus *ensembles* that are optimal from an information theoretical point of view (Machens et al., 2005). This is a computationally highly demanding task. With ever-rising computer power, however, it might be interesting to extend this concept and search for iso-information stimulus ensembles.

A prominent research area that could also benefit strongly from the iso-response methodology concerns the computations carried out by dendrites and dendritic trees. Synaptic integration along dendrites is often assumed to be linear, although it has been known for a long time that non-linearities exist and that they can have substantial consequences for neuronal computation (Koch et al., 1983; Mel, 1994; Poirazi et al., 2003; Katz et al., 2009; Abrahamsson et al., 2012). Based on traditional measurement paradigms, however, electrophysiological as well as imaging experiments can only address the question whether synaptic integration is linear, sublinear, or perhaps superlinear. Characterizing these non-linearities using the iso-response method would be an important step toward understanding dendritic computation. To investigate the scope and limits of such an approach, one could first focus on single-cell models of increasing complexity (Herz et al., 2006) with which one can test the method under well-defined and easily modifiable control conditions.

As demonstrated by the examples presented in this review, the iso-response method opens a new vista on neural dynamics and information processing. By focusing on one key question—“Which input combinations generate the *same* neural output?”—the method automatically reveals the invariance classes of the neuron (or neural substructure) under study. This feature should prove particularly helpful for studying sensory systems with complex and poorly understood stimulus spaces, such as olfaction, as well as for understanding multi-sensory integration and higher cortical processing. Note in this context that neural responses in the cortical area MST have been explained using a LNLN cascade model (Mineault et al., 2012). As shown in this review, the iso-response method is ideally suited to explore such models and determine their parameters with high precision. This suggests that even neural processing levels far from the sensory periphery can be studied quantitatively using the iso-response method.

At least conceptually, one could also extend this method beyond the single-neuron level and study multi-neuronal activity patterns. As a simple example, one may explore iso-synchrony stimuli that keep the level of synchronous activity between two or more neurons constant. Searching for multi-neuronal response patterns will require some conceptual developments regarding the applied search algorithm, that is, how to systematically tune stimuli toward eliciting a given multi-neuronal spike pattern. On the technological side, the necessary methods for fast and reliable online spike detection and sorting of multiple spike trains have already begun to become available (Quiroga et al., 2004; Santhanam et al., 2004; Wood et al., 2004; Rutishauser et al., 2006), but still need to be further explored for practical applications of closed-loop experiments.

At a larger scale, network activity could be characterized by identifying iso-population-response stimuli, using local-field-potential, MEG, or even fMRI signals. As for single neurons, one may learn far more by carefully analyzing those stimulus combinations that cause the same large-scale response than by observing that certain stimuli lead to more activation than others—without really knowing how to interpret differences in the activation levels. Within the iso-response framework, the tricky task of construing activity changes can be circumvented, and one can directly focus on one of the most important functional characteristics of a specific neuron or neural population: How are sensory or synaptic inputs integrated over space, frequencies, and time?

### Conflict of interest statement

The authors declare that the research was conducted in the absence of any commercial or financial relationships that could be construed as a potential conflict of interest.
